# The Mobile Colistin Resistance Gene, *mcr-1.1*, Is Carried on IncX4 Plasmids in Multidrug Resistant *E. coli* Isolated from Rainbow Trout Aquaculture

**DOI:** 10.3390/microorganisms8111636

**Published:** 2020-10-23

**Authors:** Jouman Hassan, Razan Zein Eddine, David Mann, Shaoting Li, Xiangyu Deng, Imad P. Saoud, Issmat I. Kassem

**Affiliations:** 1 Center for Food Safety and Department of Food Science and Technology, University of Georgia, 1109 Experiment Street, Griffin, GA 30223-1797, USA; Jouman.hassan@uga.edu (J.H.); dmann29@uga.edu (D.M.); shaoting.li25@uga.edu (S.L.); xdeng@uga.edu (X.D.); 2 Department of Biology, American University of Beirut (AUB), Beirut 1107 2020, Lebanon; rz50@aub.edu.lb (R.Z.E.); is08@aub.edu.lb (I.P.S.); 3 Department of Nutrition and Food Sciences, Faculty of Agricultural and Food Sciences, American University of Beirut, Beirut 1107 2020, Lebanon

**Keywords:** colistin, *mcr-1*, multidrug resistance, IncX4 plasmid, *E. coli*, aquaculture, Rainbow Trout, antibiotics, Lebanon

## Abstract

Colistin, a last resort antibiotic, is important for controlling infections with carbapenem-resistant *Enterobacteriaceae*. The recent emergence of mobile-colistin-resistance (*mcr*) genes has threatened the effectiveness of colistin. Aquaculture is hypothesized to be a major contributor to the evolution and dissemination of *mcr*. However, data on *mcr* in aquaculture are limited. Here, the occurrence of *mcr-1* was assessed in Rainbow Trout in Lebanon, a country with developing antimicrobial stewardship and an established use of colistin for medical and farming purposes. *mcr-1* was detected in 5 *Escherichia coli* isolated from fish guts. The isolates were classified as multidrug-resistant and their colistin minimum inhibitory concentration ranged between 16 and 32 μg/mL. Whole genome sequencing analysis showed that *mcr-1* was carried on transmissible IncX4 plasmids and that the isolates harbored more than 14 antibiotic resistance genes. The isolates belonged to ST48 and ST101, which have been associated with *mcr* and can occur in humans and fish. The *mcr-1*-positive *E. coli* persisted in 6-day biofilms, but there was a potential fitness cost. Given the status of infrastructure in Lebanon, there is a high potential for the dissemination of *mcr* via aquatic environments. Urgent actions are needed to control *mcr* and to enhance antimicrobial stewardship in Lebanon.

## 1. Introduction

Antibiotics remain a major tool in the fight against infectious diseases. However, the proliferation of antibiotic resistance has negatively impacted the efficacy of antibiotic therapy, raising significant public health concerns worldwide. Antibiotic resistance is predicted to result in severe mortalities and morbidities and economic losses which would increase the cycle of poverty, especially in developing nations [1]. Consequently, the World Health Organization (WHO) has recognized antimicrobial resistance (AMR) as an urgent and global crisis. In recent years, there has been a notable increase in resistance to last resort antibiotics, which poses a particular concern due to the importance of these drugs in treating complicated human infections [2]. Notably, the emergence and proliferation of transmissible resistance have threatened the use of colistin, a last-resort antibiotic, in controlling multidrug-resistant bacterial pathogens [3,4].

Colistin (polymyxin E) belongs to the polymyxins class of antibiotics and was designated by the WHO as one of the highest priority critically important antibiotics for human medicine [5]. Colistin was discovered in 1947 and extracted from the bacterium *Paenibacillus polymyxa* var. *colistinus*. The US FDA approved colistin for treating human infections with Gram-negative bacteria in 1959. Due to adverse effects, mainly nephrotoxicity and neurotoxicity, colistin was withdrawn and replaced by safer options such as cephalosporins. Later, colistin was reintroduced to human medicine due to limited alternatives for treating increasingly complicated infections [6] caused by carbapenem-resistant *Enterobacteriaceae* (CRE), carbapenem-resistant *Acinetobacter baumannii* (CRAB), multidrug-resistant *Pseudomonas* species, extensively drug-resistant (XDR) *Pseudomonas aeruginosa*, and XDR *Acinetobacter baumannii* [7,8]. Until recently, resistance to colistin was largely thought to occur via chromosomal mutations with restricted ability for lateral transmission between bacteria [9]. However, in 2016, a laterally transmissible plasmid-borne genetic element that conferred resistance to colistin was identified in China. The element was dubbed the mobile-colistin-resistance (*mcr-1*) gene and was detected in *Escherichia coli* isolated from pigs [3]. Since then, *mcr-1* has been reported on 5 continents and in different bacterial species and niches. Additionally, *mcr* variants and other genes (e.g., *mcr-2* to *mcr-10*) have been described [4,10,11,12,13,14,15,16,17]. The emergence and global dissemination of *mcr* have severely threatened the effectiveness of colistin, raising public health concerns globally [18]. It can be argued that the impact would be most severe in countries with poor infrastructure, developing antimicrobial stewardship, and an increasing prevalence of multi-drug resistance [18].

The rise and spread of *mcr* have been linked to the use of colistin in food animals [18,19]. Certainly, *mcr* was more frequently detected in food animals such as poultry and pigs and in food animal products in multiple nations [18]. Interestingly, it was also hypothesized that aquaculture might be a main source of dissemination of *mcr* [20,21]. It was proposed that aquatic systems are a significant reservoir for colistin-resistant genes and can transmit them directly and indirectly to humans [22]. Furthermore, seven and two *mcr-1*-positive *E. coli* were recovered from grass carp fish farms and fish in integrated fishery operations in Guangzhou in Southern China [23,24]. In Vietnam, *mcr-1* was detected in one extended-spectrum beta-lactamase (ESBL)-producing *E. coli* isolated from striped catfish grown in ponds [25,26]. A recent study from Spain reported *mcr-1* in *Salmonella enterica* serovar Rissen isolated from mussels [27]. However, beyond these few studies on farmed fish in China and Vietnam and on mussels in Spain [23,24,25,26,27], data on the occurrence of *mcr* in aquaculture have been limited. Therefore, it is important to investigate colistin resistance and *mcr* in aquaculture globally, especially in developing countries with limited infrastructure and documented challenges in pollution.

Lebanon is a Mediterranean country that faces a variety of challenges in antimicrobial stewardship, infrastructure, and pollution and is currently experiencing severe economic and political crises. In Lebanon, colistin is readily available for medical and agricultural purposes [28]. Recently, *mcr-1* was detected on food-animal farms, with high prevalence in preharvest poultry farms [29,30,31]. Furthermore, *mcr-1* was reported in clinical [32] and non-clinical settings [33], refugee camps [34,35], irrigation water [36], and seawater [37] in Lebanon. Consequently, we launched a national program to monitor the occurrence of colistin resistance and *mcr* in a variety of matrices and niches in Lebanon. Here, we focused on the analysis of *mcr* in aquaculture operations, specifically Rainbow trout farming, which is a growing industry in the Beqaa Valley of Lebanon.

## 2. Materials and Methods

### 2.1. Sample Collection and E. coli Isolation

During a national survey of surface water and agricultural systems, six-months-old fish (*n* = 6) were collected from a farm of Rainbow Trout, *Oncorhynchus mykiss*, on the Assi River (Beqaa, Lebanon). The fish were transported alive in a water cooler containing pond water with proper aeration to the laboratory. The fish were euthanized by exposure to tricaine methanesulfonate (Sigma-Aldrich, St. Louis, MO, USA) and degutted aseptically from the anus to the first ceca of the pyloric stomach. The guts were then extracted, weighed, and every two guts were pooled together. Fish guts were placed in buffered peptone water (BPW; BioRad, Hercules, CA, USA) and homogenized for 1 min in a stomacher. An aliquot (100 µL) was spread onto RAPID’ *E. coli* 2 (BioRad, Hercules, CA, USA) agar plates supplemented with colistin (4 μg/mL) (Sigma-Aldrich, St. Louis, MO, USA) [29,36], which were incubated at 37 °C. All suspected *E. coli* (violet to pink colonies) were purified, suspended in 1 mL of Luria-Bertani (LB) broth (Oxoid, Hampshire, UK) with 0.5 mL of 80% glycerol and stored at −80 °C for further analysis [29,35,36]. The experimental protocol was approved by the Animal Care and Use Committee of the American University of Beirut (AUB).

### 2.2. E. coli Identity Confirmation and Detection of mcr and Other Relevant Genes Using Polymerase Chain Reaction (PCR)

DNA was extracted from pure bacterial cultures by boiling for 15 min at 95 °C. PCR reactions were then prepared as follows: 3 µL of DNA, 0.5 µL of each of the forward and reverse specific primers, 4 µL of master mix (5× FIREPol^®^ Master Mix Ready to Load, Solis BioDyne, Tartu, Estonia), and 12 µL of DNase free water. The identity of the *E. coli* isolates was further confirmed by targeting a species-specific 16S rRNA gene fragment. The PCR analysis also included screening for *mcr-1* and other *mcr* genes (*mcr-2*, *mcr-3*, *mcr-4*, *mcr-5*, *mcr-6*, *mcr-7*, *mcr-8*) (Table 1). Reactions without DNA were used as a negative control, while DNA from a previously confirmed *mcr*-*1*-positive *E. coli* was used as a positive control [35]. The amplified products were analyzed by electrophoresis in 1% agarose gel containing ethidium bromide (BioRad, Hercules, CA, USA) and visualized using a gel imaging system (BioRad, Hercules, CA, USA). 1 Kb DNA ladder (Solis BioDyne, Tartu, Estonia) was also loaded in the gel to determine the size of the amplicons.

Using PCR analysis, the *E. coli* isolates were also screened for other genes, including *bla*_TEM-1_, *bla*_CTX-M_, *bla*_SHV-1_, *bla*_NDM-1_, *bla*_OXA-48_, *bla*_IMP_, *bla*_KPC_, *int1*, Class 1 Integron gene, Class 2 Integron gene, *tetA*, *tetB*, *tetC*, *tetD*, *tetE*, *tetG*, and *strA* (Table 1).

### 2.3. Confirmation of mcr-1 Detection Using Commercial Sequencing

The amplified gene fragments were purified using QIAquick^®^ PCR Purification Kit (QIAGEN, Germantown, MD, USA) following the manufacturer’s recommendations and sequenced commercially. The sequences were analyzed using the BlastN program and the Genbank (NCBI) database.

### 2.4. Assessment of Antimicrobial Susceptibility Using the Disk Diffusion Assay

The antibiotic resistance profiles of the *mcr-1*-positive *E. coli* were determined by the disk diffusion assay [52]. The turbidity of bacterial cultures was standardized using a 0.5 McFarland standard and a spectrophotometer. The bacterial suspensions were then spread on Mueller-Hinton agar (MH) plates (Oxoid, Hampshire, England). Eighteen commercially available antibiotic discs, including penicillin (6 µg), ampicillin (10 µg), amoxicillin/clavulanic acid (20 µg/10 µg), cefixime (5 µg), cephalexin (30 µg), cefotaxime (30 µg), cefepime (30 µg), doripenem (10 µg), meropenem (10 µg), imipenem (10 µg), gentamicin (10 µg), kanamycin (30 µg), streptomycin (10 µg), ciprofloxacin (5 µg), norfloxacin (10 µg), tetracycline (30 µg), chloramphenicol (30 µg), and trimethoprim/sulfamethoxazole (25 µg) were added to the MH plates, which were incubated at 37 °C for 18–24 h. Erythromycin (15 µg) was used as a control, because *E. coli* is intrinsically resistant to this antibiotic [53]. Furthermore, *E. coli* DH5α was used for quality control. Antimicrobial susceptibility was interpreted by measuring the diameter of the zone of inhibition and comparing it to the Clinical and Laboratory Standards Institute (CLSI) [52] and the European Committee on Antimicrobial Susceptibility Testing (EUCAST) standards [54].

### 2.5. Determination of the Colistin Minimum Inhibitory Concentration (MIC)

The minimum inhibitory concentrations (MIC) of colistin were determined for all the *mcr-1*-positive *E. coli* isolates using the broth microdilution assay [52]. Briefly, 96-well plates were inoculated with 100 µL of bacterial suspension and challenged with serially diluted colistin (Sigma-Aldrich, St. Louis, MO, USA) at concentrations ranging from 1 μg/mL to 64 μg/mL. The 96-well plates were incubated at 37 °C for 18–24 h and analyzed using a microplate reader at λ = 600 nm. Isolates with a colistin breakpoint >2 μg/mL were considered resistant as per the CLSI/EUCAST recommendations [54,55]. Wells containing MH broth and colistin only (no bacteria) and those with *E. coli* DH5α were used for quality control.

### 2.6. Assessment of the Transmissibility of mcr-1 Using Plasmid Transformation Assays

Plasmids were extracted from the *mcr-1*-positive *E. coli* using the QIAGEN^®^ Plasmid Mini Kit (QIAGEN, Germantown, MD, USA) [25] and stored at −20 °C. The plasmids were introduced to recipient chemically competent *E. coli* JM109 cells using the heat-shock method [35,36]. Briefly, 50 µL of chemically competent *E. coli* JM109 cells were mixed with 10 µL of the extracted plasmid and incubated on ice for 30 min. Competent cells with 10 µL of autoclaved deionized water (no plasmids added) were used as a control. The mixtures were then subjected to heat-shock 42 °C for 60 s in a water bath and then incubated on ice for 2 min. Freshly prepared LB broth (940 μL) was added to the mixture which was then placed in a shaking incubator (180 rpm) for 105 min at 37 °C. The mixture was then centrifuged for 2 min at 14,000 rpm and 0.9 mL of the supernatant was removed. The pellet was resuspended in the remaining 0.1 mL LB broth, and the suspension was spread onto a RAPID’ *E. coli* 2 agar plates supplemented with 2 μg/mL of colistin. The plates were incubated at 37 °C for 18–24 h. The transformants were harvested and further analyzed for the acquisition of the *mcr*-*1*, colistin MIC, and antibiotic resistance as described above [29,35].

### 2.7. Determining the DNA Fingerprint Profiles of the E. coli Isolates Using BOX-PCR

BOX-PCR typing of the *mcr-1*-positive *E. coli* was performed using the BOX-A1R primer (5′-CTACGGCAAGGCGACGCTGACG-3′) [36]. All PCR reactions (25 µL) contained 3 µL of DNA, 0.5 µL Box-A1R, 4 µL master mix (5× FIREPol^®^ Master Mix Ready to Load, Solis BioDyne, Tartu, Estonia) and 17.5 µL DNase free water. The PCR program was: an initial denaturation step for 2 min at 94 °C followed by 38 cycles, each consisting of 30 s at 94 °C followed by annealing at 50 °C for 1 min and extension for 8 min at 65 °C. The final extension step was for 8 min at 65 °C. PCR products were analyzed on 2% agarose gels containing ethidium bromide. 1 Kb DNA ladder was also loaded in the gel to compare between the BOX-PCR profiles.

### 2.8. Typing of the Plasmids in the mcr-1-Positive E. coli

The PCR based replicon typing (PBRT) kit 2.0 (Diatheva, Fano, Italy) was used to determine the plasmid types in the *mcr-1*-positive *E. coli* following the manufacturer’s recommendations [56]. The amplified PCR products were analyzed by electrophoresis for 45 min at 100 V in a 2.5% agarose gel containing ethidium bromide.

### 2.9. Assessment of Persistence of the mcr-1-Positive Isolates in Biofilms

Overnight cultures of the *mcr-1*-positive *E. coli* were diluted 1:100 and incubated shaking (200 rpm) at 37 °C for 2 h. The optical density (OD_600_) was then adjusted to 0.05. Next, 2 mL aliquots of the cultures were transferred to 5 mL sterile borosilicate vials and incubated for three and six days at 37 °C. Non-adherent bacterial cells were removed by washing with sterile distilled water. The vials were then dried and stained with 2 mL of 0.1% crystal violet stain for 15 min at room temperature. The vials were washed several times with distilled water and dried. After this, 2 mL of 30% acetic acid were added to each vial and incubated for 1 h at room temperature. The optical density (OD) of the suspension was measured at λ = 570 nm using a spectrophotometer. Vials containing LB broth only were used as negative controls. Samples were tested in duplicates and the biofilm experiments were repeated on three separate occasions.

The persistence of the *mcr-1* in isolates retrieved from 3- and 6-day biofilms was also tested. Biofilms were setup as described above. However, after non-adherent bacteria were removed by washing with sterile distilled water, the biofilms were resuspended in 1 mL of LB broth and serially diluted (10-fold). After this, 100 μL of the diluted aliquots were spread on RAPID’ *E. coli* 2 agar plates without and with colistin (4 μg/mL). The plates were incubated at 37 °C for 18–24 h and colony forming units (CFU) were counted. *E. coli* growing on colistin containing plates were tested for *mcr-1* using PCR analysis as described above. Samples were analyzed in duplicates and the experiments were repeated on three separate occasions.

### 2.10. Whole Genome Sequencing (WGS) Analysis of mcr-1-Positive E. coli

Based on the BOX-PCR profiles and the antibiotic resistance properties, DNA was extracted from two representative *mcr-1*-positive isolates using the QiaAmp DNA Mini kit (Qiagen, Germantown, MD, USA) following the manufacturer’s recommendations. DNA concentrations were determined by the Qubit BR dsDNA assay kit (Invitrogen, Waltham, MA, USA) and diluted to 0.2 ng/μL. Libraries were prepared using the Illumina Nextera XT DNA Library preparation kit (Illumina, San Diego, CA, USA) [57]. The Qubit dsDNA HS assay kit (Invitrogen, Waltham, MA, USA) was used to determine the concentration of the sample libraries, which were then diluted to 2 nM and combined in equal volumes to form a pooled library. Six hundred μL of the denatured pooled library (10 pM) were loaded into the MiSeq reagent cartridge (MiSeq reagent kit v2, 300 cycles) and sequenced using a MiSeq sequencer (Illumina, San Diego, CA, USA) [57]. The sequence reads were trimmed to remove low-quality reads using Trimmomatic v0.36 [58]. Spades v3.9.0 was used to assemble the filtered and trimmed reads using the “–careful” option [59]. Evaluation of the genome quality and the N50 values were determined by QUAST v4.5. The ResFinder database (v3.0) [60] was used to identify the antimicrobial resistance genes in the isolates. The PlasmidFinder database (v1.3) [61] was used to determine the plasmid type that carried the *mcr-1*. Sequence types (STs) were determined using the assembled genomes and the PubMLST database (https://pubmlst.org/) with MLST software (https://github.com/tseemann/mlst) [62].

### 2.11. Data Availability

Whole-genome sequences for F1 and F2 strains were deposited in GenBank under accession numbers: SRX7741078 and SRX7741079, respectively.

## 3. Results and Discussion

Five typical (violet to pink color) *E. coli* colonies were detected in the sample that contained the pooled guts of fish #1 and #4. The identity of the isolates was further confirmed using PCR analysis that targeted an *E. coli*-species-specific 16S-rRNA gene fragment [29,35,36] (Table 1). The *E. coli* were positive for *mcr-1* and negative for other *mcr* genes (*mcr-2* to *8*), which was confirmed by commercial sequencing of the PCR amplicons. The colistin minimum inhibitory concentration (MIC) of the isolates ranged between 16 and 32 μg/mL (Table 2), indicating further that the strains were colistin resistant. Additional analysis showed that the *mcr-1* was successfully introduced to chemically competent *E. coli* JM-109 (a colistin-susceptible and *mcr-1*-negative strain), which revealed that the gene was plasmid-born [29,35,36]. Specifically, the transformants were colistin-resistant (MIC = 4–8 μg/mL) and were found to carry *mcr*-*1* using PCR analysis.

**Figure 1:**
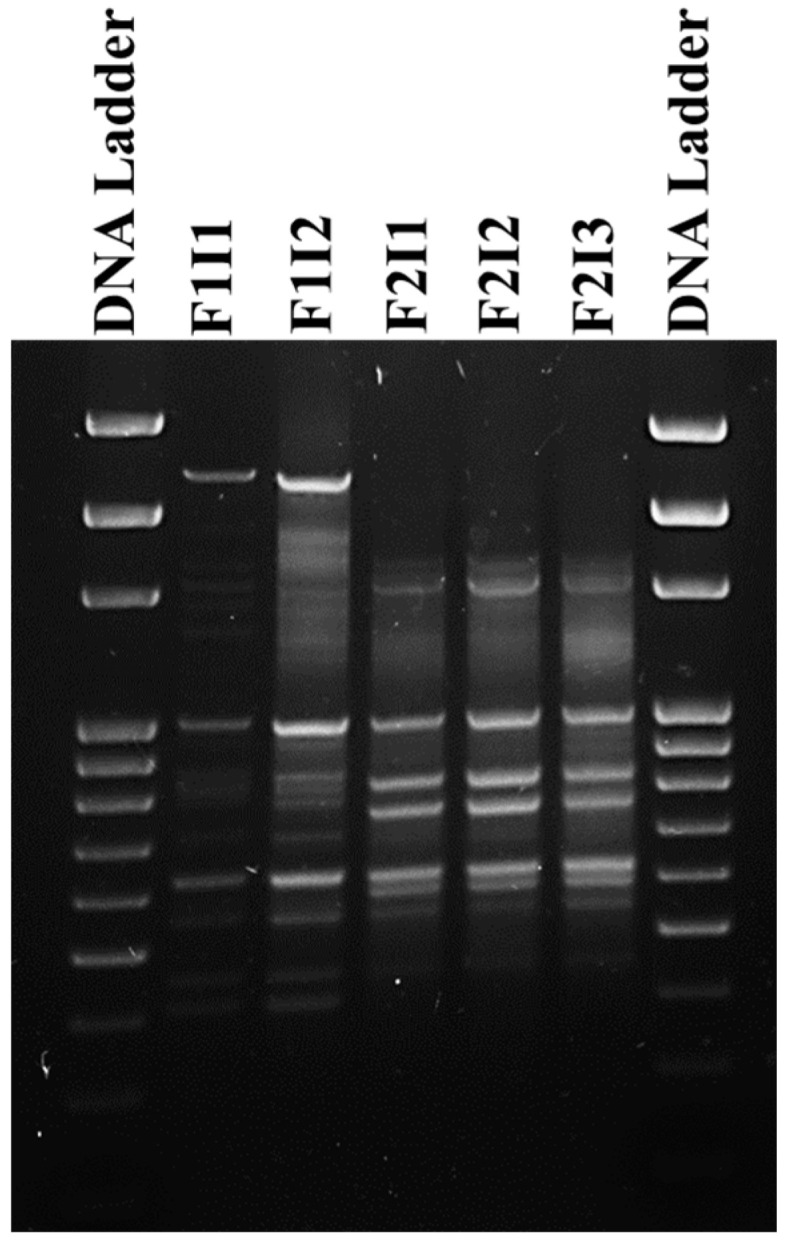
BOX-PCR analysis of the *mcr-1*-positive *E. coli*. The analysis revealed that the 5 isolates belonged to two distinct fingerprint profiles that were classified as F1 and F2. Isolates are designated by the letter I.

Preliminary typing using BOX-PCR analysis divided the isolates (at 100% similarity) into two genotypes that were designated F1 (*n* = 2) and F2 (*n* = 3) (Figure 1). The disk diffusion assay showed that F1 and F2 isolates were multidrug-resistant (resistance >3 antibiotic classes) (Table 2). All F1 (F1I1 and F1I2) isolates exhibited resistance to penicillin (PEN), ampicillin (AMP), amoxicillin/clavulanate (AMC), cephalexin (LEX), gentamicin (GEN), kanamycin (KAN), streptomycin (STR), tetracycline (Tet), trimethoprim/sulfamethoxazole (SXT), and chloramphenicol (CHL), while all F2 (F2I1, F2I2, and F2I3) isolates were resistant to PEN, AMP, AMC, LEX, KAN, STR, TET, ciprofloxacin (CIP), norfloxacin (NOR), SXT, and CHL (Table 2). Subsequently, the AMR profile analysis confirmed that the isolates can be divided into two groups, because isolates in each group had identical AMR phenotypes. Furthermore, PCR analysis showed that all the isolates were positive to class I integron genes and the *int1* (integrase encoding gene) but not class 2 integron genes. This is important, because class I integrons provide the ability to acquire, express and laterally transfer different antibiotic resistance genes [63], which highlighted the ability of the *mcr-1*- positive *E. coli* isolated from fish to be multidrug resistant and supported the aforementioned AMR phenotype analysis. PCR analysis also showed that the isolates were negative for *bla*_CTX-M_, *bla*_SHV-1_, *bla*_NDM-1_, *bla*_OXA-48_, *bla*_IMP_, *bla*_KPC_, *tetB*, *tetC*, *tetD*, *tetE*, *tetG* and positive for *bla*_TEM-1_ and *tetA*, while *strA* was only detected in the F2 isolates (Table 2). This again corroborated the AMR phenotypes of the isolates.

MLST analysis of the sequenced genomes showed that F1 and F2 belonged to sequence types, ST48 and ST101, respectively. These STs have been associated with *mcr* and multidrug resistant strains and occur in a variety of niches and hosts, including humans, avian hosts, and fish [23,64,65,66]. This highlighted the ability of *mcr-1* to spread to important matrices and hosts and suggested that F1 and F2 isolates can be transmitted beyond the trout farm and/or can be acquired by the fish from other polluted sources [64].

Using the ResFinder database (v3.0) [60], WGS revealed that the isolates carried *mcr-1.1* specifically. Additionally, F1 and F2 harbored more than 14 antibiotic resistance genes (Table 2). The genes encoded resistance to aminoglycosides, diaminopyrimidines, macrolides, streptogramins, lincosamides, phenicols, fosfomycin, tetracyclines, macrolides, fluoroquinolones, and sulfonamides, highlighting the multidrug resistant properties of these isolates (Table 2). Using PlasmidFinder (v1.3) [61], *mcr-1.1* was shown to be carried on IncX4 plasmids, which have been associated with the global spread of this gene [4,65]. Additionally, IncX4 plasmids are known to be prevalent in *E. coli*, occur in other *Enterobacteriaceae*, and exhibit high transmission frequency [65,67]. This plasmid type and the AMR genes detected by WGS were corroborated using PBRT typing [56] and PCR analysis that targeted a subset of the genes.

**Figure 2:**
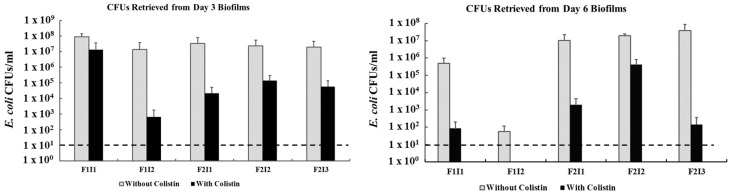
The persistence of *mcr-1*- positive *E. coli* in 3- and 6 days old biofilms. The grey and black colors designate CFUs retrieved on RAPID’ *E. coli* 2 agar plates without and with colistin (4 μg/mL), respectively. The dashed line designates the detection limit of the assay.

Notably, *mcr-1.1* persisted in colony forming units retrieved from 3- and 6-day-old biofilms under aerobic conditions. However, there was ~2–3 log and up to 5 log reduction in colistin resistant CFUs retrieved from day 3 and 6 biofilms, respectively (Figure 2). The reduction differed between isolates and was most pronounced in day 6 biofilms for all isolates except F2I2 (Figure 2). This suggested that *mcr-1.1* can persist in biofilms at least till day 6. However, a population of the *E. coli* in the biofilms lost colistin resistance, perhaps highlighting a previously undescribed fitness cost associated with *mcr-1.1*-carrying IncX4 plasmids.

In a study conducted on integrated fish-duck farming, it was suggested that *E. coli* harboring the *mcr-1* can spread from aquatic animals to affect the supply chain and humans [24]. Therefore, the detection of *mcr-1.1* in fish poses a significant public health concern, because resistance can be potentially transmitted to pathogens that affect humans and other animals [23,24]. Additionally, international trade with farmed fish can act as a route for the dissemination of *mcr* between countries [18,68]. Here, we report the detection of transmissible *mcr-1.1* in multidrug resistant *E. coli* isolated from farmed Rainbow Trout. To our knowledge, this report is one of a few studies on *mcr-1.1* in aquaculture and the first to document this gene in farmed Rainbow Trout worldwide and in aquaculture in the Middle East and North Africa (MENA) region. It should be noted that the number of tested fish samples was limited, which restricts the extrapolation of the findings to all aquaculture farms in Lebanon. Furthermore, the source of the *mcr-1*-positive *E. coli* in the Trout is currently unclear. However, there is ample evidence to suggest that aquatic environments in Lebanon are affected by anthropogenic waste, including sewage and agricultural and industrial contaminants. Indeed, previous studies have detected *mcr-1*-positive *E. coli* in Lebanese poultry, irrigation water, and other niches, while colistin is widely available in medical and agricultural practices in this country [29,30,31,32,33,34,35,36,37]. Therefore, there is opportunity for the spread of *mcr*-*1.1* to different matrices and hosts in Lebanon via cross-contamination. This jeopardizes the control of infectious diseases and can potentially result in the proliferation of resistance to colistin in vital resources locally and beyond [37]. Therefore, we emphasize an urgent need for investment in antimicrobial stewardship and AMR surveillance and outreach in Lebanon and other countries with similar challenges. Given that Lebanon is currently experiencing the worst economic crisis in its recent history, we call for global action to address the spread of AMR in Lebanon, which will benefit the region as well as the global fight against AMR.

## Figures and Tables

**Table 1:** Primers and polymerase chain reaction (PCR) conditions used for screening the *E. coli* isolates for *mcr* genes and other relevant AMR genes.

Targeted Gene/Fragment	Primers	Primer Sequences (5′-3′)	Amplicon Size (bp)	Reference
*E. coli* specific 16S r-RNA gene	16S r-RNA-F	AAGAAGCTTGCTTCTTTGCTGAC	544	[38]
	16S r-RNA-R	AGCCCGGGGATTTCACATCTGACTTA		
*mcr-1*	CLR5-F	CGGTCAGTCCGTTTGTTC	309	[3]
	CLR5-R	CTTGGTCGGTCTGTA GGG		
*mcr-2*	*mcr*-2F	GCGATGGCGGTCTATCCTGTAT	378	[39]
	*mcr*-2R	TGCGATGACATGGGGTGTCAGC		
*mcr-3*	*mcr*-3F	TATGGGTTACTATTGCTGG	814	
	*mcr*-3R	CTACCCTGATGCTCATCG		
*mcr-4*	*mcr*-4F	GTCATAGTGGTATAAAAGTACAG	664	
	*mcr*-4R	CCACCGTCTATCAGAGCCAAC		
*mcr-5*	*mcr*-5F	GCGGTTGTCTGCATTTATCAC	1042	
	*mcr*-5R	CTTTGAAAACCTGTCTTCGGCA		
*mcr-6*	*mcr*-6F	GTCCGGTCAATCCCTATCTGT	556	
	*mcr*-6R	ATCACGGGATTGACATAGCTAC		
*mcr-7*	*mcr*-7F	TGCTCAAGCCCTTCTTTTCGT	892	
	*mcr*-7R	TTCATCTGCGCCACCTCGT		
*mcr-8*	*mcr*-8F	AACCGCCAGAGCACAGAATT	667	
	*mcr*-8R	TTCCCCCAGCGATTCTCCAT		
*bla*_TEM-1_	*bla*_TEM-1_-F	ACCAATGCTTAATCAGTGAG	963	[40]
	*bla*_TEM-1_-R	GCGGAACCCCTATTTG		
*bla*_CTX-M_	*bla*_CTX-M_-F	ATGTGCAGYACCAGTAARGTKATGGC	593	[41]
	*bla*_CTX-M_-R	TGGGTRAARTARGTSACCAGAAYCAGCGG		
*bla*_SHV_	*bla*_SHV_-_1_-F	CACTCAAGGATGTATTGTG	822	[42]
	*bla*_SHV_-_1_-R	TTAGCGTTGCCAGTGCTCG		
*bla*_NDM-1_	*bla*_NDM_-_1_-F	GGTTTGGCGATCTGGTTTTC	621	[43]
	*bla*_NDM-1_-R	CGGAATGGCTCATCACGATC		
*bla*_OXA-48_	*bla*_OXA-48_-F	GCTTGATCGCCCTCGATT	238	[44]
	*bla*_OXA-48_-R	GATTTGCTCCGTGGCCGAAA		
*bla*_IMP_	*bla*_IMP_-F	TGAGCAAGTTATCTGTATTC	740	[45]
	*bla*_IMP_-R	TTAGTTGCTTGGTTTTGATG		
*bla*_KPC_	*bla*_KPC_-F	CATTCAAGGGCTTTCTTGCTGC	498	[44]
	*bla*_KPC_-R	ACGACGGCATAGTCATTTGC		
*int1*	*int1*-F	CCTCCCGCACGATGATC	270	[46]
	*int1*-R	TCCACGCATCGTCAGGC		
Class 1 Integron gene	Class 1 Integron gene-F	GGCATCCAAGCACAAGC	Variable	[47]
	Class 1 Integron gene-R	AAGCAGACTTGACTGAT		
Class 2 Integron gene	Class 2 Integron gene-F	GGGATCCCGGACGGCATGCACGATTTGTA	Variable	[48]
	Class 2 Integron gene-R	GATGCCATCGCAAGTACGA		
*tetA*	*tetA*-F	GTAATTCTGAGCACTGTCGC	950	[49]
	*tetA*-R	CTGCCTGGACAACATTGCTT		
*tetB*	*tetB*-F	CTCAGTATTCCAAGCCTTTG	430	
	*tetB*-F	ACTCCCCTGAGCTTGAGGGG		
*tetC*	*tetC*-F	GGTTGAAGGCTCTCAAGGGC	505	
	*tetC*-R	CCTCTTGCGGGATATCGTCC		
*tetD*	*tetD*-F	CATCCATCCGGAAGTGATAGC	435	
	*tetD*-R	CATCCATCCGGAAGTGATAGC		
*tetE*	*tetE*-F	TGATGATGGCACTGGTCA	262	[50]
	*tetE*-R	GCTGGCTGTTGCCATTA		
*tetG*	*tetG*-F	GCAGCGAAAGCGTATTTGCG	680	[49]
	*tetG*-R	TCCGAAAGCTGTCCAAGCAT		
*strA*	*strA*-F	CCT ATC GGT TGA TCA ATG TC	250	[51]
	*strA*-R	GAAGAGTTTTAGGGTCCACC		

**Table 2:** Antibiotic resistance phenotypes and antimicrobial resistance (AMR) genes of the *mcr-1*-positive and colistin-resistant *E. coli* isolated from Rainbow Trout in Lebanon. Penicillin (PEN), ampicillin (AMP), amoxicillin + clavulanic acid (AMC), cephalexin (LEX), gentamicin (GEN), kanamycin (KAN), streptomycin (STR), tetracycline (TET), ciprofloxacin (CIP), norfloxacin (NOR), trimethoprim-sulfamethoxazole (SXT), chloramphenicol (CHL), (MIC) minimum inhibitory concentration.

Location	Fish Species	*E. coli* ID Codes	Antibiotic Resistance Profiles of Colistin Resistant *E. coli*	Colistin MIC (μg/mL)	AMR Genes Detected by WGS and PCR Analysis ^1^	Sequence Types
Beqaa Valley (Hermel)	Rainbow Trout (*Oncorhynchus mykiss*)	F1I1	PEN-AMP-AMC-LEX-GEN-KAN-STR-TET-SXT-CHL	16	*mcr-1.1*, *aac(3)-IId*, *aadA2*, *ant(3″)-Ia*, *aph(3′)-Ia*, *bla_TEM-1B_*, *dfrA12*, *erm42*, *floR*, *mdf(A)*, *mph(A)*, *sul1*, *sul2*, *tetA*,	ST48
		F1I2	PEN-AMP-AMC-LEX-GEN-KAN-STR-TET-SXT-CHL	16		
		F2I1	PEN-AMP-AMC-LEX-KAN-STR-TET-CIP-NOR-SXT-CHL	32	*mcr-1.1*, *aph(3″)-Ib*, *aph(3′)-Ia*, *aph(6)-Id*, *bla_TEM-1_*, *dfrA14*, *floR*, *fosA3*, *mdf(A)*, *mph(A)*, *qnrS1*, *strA*, *sul2*, *tetA*, *tetM*,	ST101
		F2I2	PEN-AMP-AMC-LEX-KAN-STR-TET-CIP-NOR-SXT-CHL	32		
		F2I3	PEN-AMP-AMC-LEX-KAN-STR-TET-CIP-NOR-SXT-CHL	32		

^1^ A subset of the genes was also detected by PCR analysis as described in the text.

## References

[1] Aslam B., Wang W., Arshad M.I., Khurshid M., Muzammil S., Rasool M.H., Nisar M.A., Alvi R.F., Aslam M.A., Qamar M.U. (2018). Antibiotic resistance: A rundown of a global crisis. Infect. Drug Resist..

[2] Ventola C.L. (2015). The antibiotic resistance crisis: Part 1: Causes and threats. Pharm. Ther..

[3] Liu Y.-Y., Wang Y., Walsh T.R., Yi L.-X., Zhang R., Spencer J., Doi Y., Tian G., Dong B., Huang X. (2016). Emergence of plasmid-mediated colistin resistance mechanism MCR-1 in animals and human beings in China: A microbiological and molecular biological study. Lancet Infect. Dis..

[4] Wang R., Van Dorp L., Shaw L.P., Bradley P., Wang Q., Wang X., Jin L., Zhang Q., Liu Y., Rieux A. (2018). The global distribution and spread of the mobilized colistin resistance gene *mcr-1*. Nat. Commun..

[5] WHO (2017). Critically Important Antimicrobials for Human Medicine: Ranking of Antimicrobial Agents for Risk Management of Antimicrobial Resistance Due to Non-Human Use.

[6] Poirel L., Jayol A., Nordmann P. (2017). Polymyxins: Antibacterial activity, susceptibility testing, and resistance mechanisms encoded by plasmids or chromosomes. Clin. Microbiol. Rev..

[7] Eichenberger E.M., Thaden J.T. (2019). Epidemiology and mechanisms of resistance of extensively drug resistant Gram-negative bacteria. Antibiotics.

[8] Van Duin D., Paterson D.L. (2016). Multidrug-resistant bacteria in the community. Infect. Dis. Clin. N. Am..

[9] Olaitan A.O., Morand S., Rolain J.-M. (2014). Mechanisms of polymyxin resistance: Acquired and intrinsic resistance in bacteria. Front. Microbiol..

[10] AbuOun M., Stubberfield E.J., Duggett N.A., Kirchner M., Dormer L., Nunez-Garcia J., Randall L.P., Lemma F., Crook D.W., Teale C. (2017). *mcr-1* and *mcr-2* (*mcr-6.1*) variant genes identified in *Moraxella* species isolated from pigs in Great Britain from 2014 to 2015. J. Antimicrob. Chemother..

[11] Borowiak M., Fischer J., Hammerl J.A., Hendriksen R.S., Szabo I., Malorny B. (2017). Identification of a novel transposon-associated phosphoethanolamine transferase gene, *mcr-5*, conferring colistin resistance in d-tartrate fermenting *Salmonella enterica* subsp. *enterica* serovar Paratyphi B. J. Antimicrob. Chemother..

[12] Carattoli A., Villa L., Feudi C., Curcio L., Orsini S., Luppi A., Pezzotti G., Magistrali C.F. (2017). Novel plasmid-mediated colistin resistance *mcr-4* gene in *Salmonella* and *Escherichia coli*, Italy 2013, Spain and Belgium, 2015 to 2016. Eurosurveillance.

[13] Carroll L.M., Gaballa A., Guldimann C., Sullivan G., Henderson L.O., Wiedmann M. (2019). Identification of novel mobilized colistin resistance gene *mcr-9* in a multidrug-resistant, colistin-susceptible *Salmonella enterica* serotype Typhimurium isolate. mBio.

[14] Ling Z., Yin W., Li H., Zhang Q., Wang X., Wang Z., Ke Y., Wang Y., Shen J. (2017). Chromosome-mediated *mcr-3* variants in *Aeromonas veronii* from chicken meat. Antimicrob. Agents Chemother..

[15] Xavier B.B., Lammens C., Ruhal R., Kumar-Singh S., Butaye P., Goossens H., Malhotra-Kumar S. (2016). Identification of a novel plasmid-mediated colistin-resistance gene, *mcr-2*, in *Escherichia coli*, Belgium, June 2016. Eurosurveillance.

[16] Yang Y.-Q., Li Y.-X., Lei C.-W., Zhang A.-Y., Wang H.-N. (2018). Novel plasmid-mediated colistin resistance gene *mcr-7.1* in *Klebsiella pneumoniae*. J. Antimicrob. Chemother..

[17] Wang C., Feng Y., Liu L., Wei L., Kang M., Zong Z. (2020). Identification of novel mobile colistin resistance gene *mcr-10*. Emerg. Microbes Infect..

[18] Hassan J., El-Gemayel L., Bashour I., Kassem I.I. (2020). On the edge of a precipice: The global emergence and dissemination of plasmid-borne *mcr* genes that confer resistance to colistin, a last-resort antibiotic. Antibiotics and Antimicrobial Resistance Genes in the Environment.

[19] Kempf I., Jouy E., Chauvin C. (2016). Colistin use and colistin resistance in bacteria from animals. Int. J. Antimicrob. Agents.

[20] Cabello F.C., Tomova A., Ivanova L., Godfrey H.P. (2017). Aquaculture and *mcr* colistin resistance determinants. mBio.

[21] Cabello F.C., Godfrey H.P. (2018). Aquaculture, exaptation, and the origin of *mcr*-positive colistin resistance. Antimicrob. Agents Chemother..

[22] Cabello F.C., Godfrey H.P. (2016). Comment on: Transferable resistance to colistin: A new but old threat. J. Antimicrob. Chemother..

[23] Lv L., Cao Y., Yu P., Huang R., Wang J., Wen Q., Zhi C., Zhang Q., Liu J.-H. (2018). Detection of *mcr-1* gene among *Escherichia coli* isolates from farmed fish and characterization of *mcr-1* -bearing IncP plasmids. Antimicrob. Agents Chemother..

[24] Shen Y., Lv Z., Yang L., Liu D., Ou Y., Xu C., Liu W., Yuan D., Hao Y., He J. (2019). Integrated aquaculture contributes to the transfer of *mcr-1* between animals and humans via the aquaculture supply chain. Environ. Int..

[25] Hoa T., Nakayama T., Huyen H.M., Harada K., Hinenoya A., Phuong N.T., Yamamoto Y., Tran H.T.T. (2019). Extended-spectrum beta-lactamase-producing *Escherichia coli* harbouring *sul* and *mcr-1* genes isolates from fish gut contents in the Mekong Delta, Vietnam. Lett. Appl. Microbiol..

[26] Yamaguchi T., Kawahara R., Harada K., Teruya S., Nakayama T., Motooka D., Nakamura S., Nguyen P.D., Kumeda Y., Van Dang C. (2018). The presence of colistin resistance gene *mcr-1* and *-3* in ESBL producing *Escherichia coli* isolated from food in Ho Chi Minh City, Vietnam. FEMS Microbiol. Lett..

[27] Lozano-Leon A., Garcia-Omil C., Dalama J., Rodriguez-Souto R., Martinez-Urtaza J., Gonzalez-Escalona N. (2019). Detection of colistin resistance *mcr-1* gene in *Salmonella enterica* serovar Rissen isolated from mussels, Spain, 2012 to 2016. Eurosurveillance.

[28] Kassem I.I., Hijazi M.A., Saab R. (2019). On a collision course: The availability and use of colistin-containing drugs in human therapeutics and food-animal farming in Lebanon. J. Glob. Antimicrob. Resist..

[29] Hmede Z., Kassem I.I. (2018). The colistin resistance gene, *mcr-1*, is prevalent in commensal *E. coli* isolated from Lebanese pre-harvest poultry. Antimicrob. Agents Chemother..

[30] Dandachi I., Leangapichart T., Daoud Z., Rolain J.-M. (2018). First detection of *mcr-1* plasmid-mediated colistin-resistant *Escherichia coli* in Lebanese poultry. J. Glob. Antimicrob. Resist..

[31] Dandachi I., Fayad E., Sleiman A., Daoud Z., Rolain J.-M. (2020). Dissemination of multidrug-resistant and *mcr-1* gram-negative bacilli in broilers, farm workers, and the surrounding environment in Lebanon. Microb. Drug Resist..

[32] Al-Mir H., Osman M., Azar N., Madec J.-Y., Hamze M., Haenni M. (2019). Emergence of clinical *mcr-1*-positive *Escherichia coli* in Lebanon. J. Glob. Antimicrob. Resist..

[33] Hmede Z., Kassem I.I. (2019). First report of the plasmid-borne colistin resistance gene (*mcr-1*) in *Proteus mirabilis* isolated from a toddler in non-clinical settings. IDCases.

[34] Sulaiman A.A.A., Kassem I.I. (2020). First report of the plasmid-borne colistin resistance gene (*mcr-1*) in *Proteus mirabilis* isolated from domestic and sewer waters in Syrian refugee camps. Travel Med. Infect. Dis..

[35] Sulaiman A.A.A., Kassem I.I. (2019). First report on the detection of the plasmid-borne colistin resistance gene *mcr-1* in multi-drug resistant *E. coli* isolated from domestic and sewer waters in Syrian refugee camps in Lebanon. Travel Med. Infect. Dis..

[36] Hmede Z., Sulaiman A.A.A., Jaafar H., Kassem I.I. (2019). Emergence of plasmid-borne colistin resistance gene *mcr-1* in multidrug-resistant *Escherichia coli* isolated from irrigation water in Lebanon. Int. J. Antimicrob. Agents.

[37] Sourenian T., Mann D., Li S., Deng X., Jaafar H., Kassem I.I. (2020). Dissemination of multidrug-resistant *Escherichia coli* harboring the mobile colistin resistance gene *mcr-1.1* on transmissible plasmids in the Mediterranean Sea. J. Glob. Antimicrob. Resist..

[38] Sabat G., Rose P., Hickey W.J., Harkin J.M. (2000). Selective and sensitive method for PCR amplification of *Escherichia coli* 16S rRNA genes in soil. Appl. Environ. Microbiol..

[39] Yang F., Shen C., Zheng X., Liu Y., Ahmed M.A.E.-G.E.-S., Zhao Z., Liao K., Shi Y., Guo X., Zhong R. (2019). Plasmid-mediated colistin resistance gene *mcr-1* in *Escherichia coli* and *Klebsiella pneumoniae* isolated from market retail fruits in Guangzhou, China. Infect. Drug Resist..

[40] Olesen I., Hasman H., Aarestrup F.M. (2004). Prevalence of β-lactamases among ampicillin-resistant *Escherichia coli* and *Salmonella* isolated from food animals in Denmark. Microb. Drug Resist..

[41] Hasman H., Mevius D., Veldman K., Olesen I., Aarestrup F.M. (2005). β-Lactamases among extended-spectrum β-lactamase (ESBL)-resistant *Salmonella* from poultry, poultry products and human patients in The Netherlands. J. Antimicrob. Chemother..

[42] Pitout J.D.D., Thomson K.S., Hanson N.D., Ehrhardt A.F., Moland E.S., Sanders C.C. (1998). β-Lactamases responsible for resistance to expanded-spectrum cephalosporins in *Klebsiella pneumoniae*, *Escherichia coli*, and *Proteus mirabilis* isolates recovered in South Africa. Antimicrob. Agents Chemother..

[43] Poirel L., Walsh T.R., Cuvillier V., Nordmann P. (2011). Multiplex PCR for detection of acquired carbapenemase genes. Diagn. Microbiol. Infect. Dis..

[44] Mushi M.F., Mshana S.E., Imirzalioglu C., Bwanga F. (2014). Carbapenemase genes among multidrug resistant Gram negative clinical isolates from a tertiary hospital in Mwanza, Tanzania. BioMed Res. Int..

[45] Zafer M.M., Al-Agamy M.H., El-Mahallawy H.A., Amin M.A., Ashour M.S.E.-D. (2014). Antimicrobial resistance pattern and their beta-lactamase encoding genes among *Pseudomonas aeruginosa* strains isolated from cancer patients. BioMed Res. Int..

[46] Goldstein C., Lee M.D., Sanchez S., Hudson C., Phillips B., Register B., Grady M., Liebert C., Summers A.O., White D.G. (2001). Incidence of class 1 and 2 integrases in clinical and commensal bacteria from livestock, companion animals, and exotics. Antimicrob. Agents Chemother..

[47] Zhao S., Fedorka-Cray P.J., Friedman S., McDermott P.F., Walker R., Qaiyumi S., Foley S., Hubert S., Ayers S., English L. (2005). Characterization of *Salmonella typhimurium* of animal origin obtained from the National Antimicrobial Resistance Monitoring System. Foodborne Pathog. Dis..

[48] Kiiru J., Butaye P., Goddeeris B.M., Kariuki S. (2013). Analysis for prevalence and physical linkages amongst integrons, ISE cp 1, IS CR 1, Tn 21 and Tn 7 encountered in *Escherichia coli* strains from hospitalized and non-hospitalized patients in Kenya during a 19-year period (1992–2011). BMC Microbiol..

[49] Rahmani M., Peighambari S.M., Svendsen C.A., Cavaco L.M., Agersø Y., Hendriksen R.S. (2013). Molecular clonality and antimicrobial resistance in *Salmonella enterica* serovars Enteritidis and Infantis from broilers in three Northern regions of Iran. BMC Vet. Res..

[50] Wilkerson C., Samadpour M., van Kirk N., Roberts M.C. (2004). Antibiotic resistance and distribution of tetracycline resistance genes in Escherichia coli O157: H7 isolates from humans and bovines. Antimicrob. Agents Chemother..

[51] Faldynova M., Pravcova M., Šišák F., Havlíčková H., Kolackova I., Cizek A., Karpíšková R., Rychlik I. (2003). Evolution of antibiotic resistance in *Salmonella enterica* Serovar Typhimurium strains isolated in the Czech Republic between 1984 and 2002. Antimicrob. Agents Chemother..

[52] Clinical and Laboratory Standards Institute (CLSI) (2016). Performance Standards for Antimicrobial Susceptibility Testing.

[53] Nguyen M.C.P., Woerther P.-L., Bouvet M., Andremont A., Leclercq R., Canu A. (2009). *Escherichia coli* as reservoir for macrolide resistance genes. Emerg. Infect. Dis..

[54] European Committee on Antimicrobial Susceptibility Testing (2018). Breakpoint Tables for Interpretation of MICs and Zone Diameters. EUCAST, Version 8.1. http://www.eucast.org.

[55] European Committee on Antimicrobial Susceptibility Testing (2016). Recommendations for MIC Determination of Colistin (Polymyxin E) as Recommended by the Joint CLSI-EUCAST Polymyxin Breakpoints Working Group.

[56] Carloni E., Andreoni F., Omiccioli E., Villa L., Magnani M., Carattoli A. (2017). Comparative analysis of the standard PCR-Based Replicon Typing (PBRT) with the commercial PBRT-KIT. Plasmid.

[57] Hyeon J.-Y., Li S., Mann D.A., Zhang S., Li Z., Chen Y., Deng X. (2018). Quasimetagenomics-based and real-time-sequencing-aided detection and subtyping of *Salmonella enterica* from food samples. Appl. Environ. Microbiol..

[58] Ondov B.D., Treangen T.J., Melsted P., Mallonee A.B., Bergman N.H., Koren S., Phillippy A.M. (2016). Mash: Fast genome and metagenome distance estimation using MinHash. Genome Biol..

[59] Bankevich A., Nurk S., Antipov D., Gurevich A.A., Dvorkin M., Kulikov A.S., Lesin V.M., Nikolenko S.I., Pham S., Prjibelski A.D. (2012). SPAdes: A new genome assembly algorithm and its applications to single-cell sequencing. J. Comput. Biol..

[60] Zankari E., Hasman H., Cosentino S., Vestergaard M., Rasmussen S., Lund O., Aarestrup F.M., Larsen M.V. (2012). Identification of acquired antimicrobial resistance genes. J. Antimicrob. Chemother..

[61] Carattoli A., Zankari E., García-Fernández A., Larsen M.V., Lund O., Villa L., Aarestrup F.M., Hasman H. (2014). In Silico Detection and typing of plasmids using PlasmidFinder and Plasmid Multilocus sequence typing. Antimicrob. Agents Chemother..

[62] Larsen M.V., Cosentino S., Rasmussen S., Friis C., Hasman H., Marvig R.L., Jelsbak L., Sicheritz-Pontén T., Ussery D.W., Aarestrup F.M. (2012). Multilocus sequence typing of total-genome-sequenced bacteria. J. Clin. Microbiol..

[63] Gillings M.R., Boucher Y., Labbate M., Holmes A., Krishnan S., Holley M., Stokes H.W. (2008). The evolution of class 1 integrons and the rise of antibiotic resistance. J. Bacteriol..

[64] Zurfluh K., Nüesch-Inderbinen M., Klumpp J., Poirel L., Nordmann P., Stephan R. (2017). Key features of *mcr-1*-bearing plasmids from *Escherichia coli* isolated from humans and food. Antimicrob. Resist. Infect. Control..

[65] Fernandes M.R., McCulloch J.A., Vianello M.A., Moura Q., Pérez-Chaparro P.J., Esposito F., Sartori L., Dropa M., Matté M.H., Lira D.P. (2016). First report of the globally disseminated IncX4 plasmid carrying the *mcr-1* gene in a colistin-resistant *Escherichia coli* sequence type 101 isolate from a human infection in Brazil. Antimicrob. Agents Chemother..

[66] Zhuge X., Ji Y., Tang F., Sun Y., Jiang M., Hu W., Wu Y., Xue F., Ren J., Zhu W. (2019). Population structure and antimicrobial resistance traits of avian-origin *mcr-1* -positive *Escherichia coli* in Eastern China, 2015 to 2017. Transbound. Emerg. Dis..

[67] Sun J., Fang L.-X., Wu Z., Deng H., Yang R.-S., Li X.-P., Li S.-M., Liao X.-P., Feng Y., Liu Y.-H. (2017). Genetic analysis of the IncX4 plasmids: Implications for a unique pattern in the *mcr-1* acquisition. Sci. Rep..

[68] Hassan J., Kassem I.I. (2020). Audacious Hitchhikers: The Role of Travel and the International Food Trade in the Global Dissemination of Mobile Colistin-Resistance (*mcr*) Genes. Antibiotics.

